# Large granular lymphocytosis after transplantation

**DOI:** 10.18632/oncotarget.21009

**Published:** 2017-09-18

**Authors:** Zhi-Yuan Qiu, Guang-Yu Tian, Zhao Zhang, Ye-Qing Zhang, Wei Xu, Jian-Yong Li

**Affiliations:** ^1^ Department of Oncology, The Affiliated People's Hospital of Jiangsu University, Zhenjiang 212002, Jiangsu, China; ^2^ Department of Vascular Surgery, The Second Affiliated Hospital of Soochow University, Suzhou 215004, Jiangsu, China; ^3^ Department of Hematology, The First Affiliated Hospital of Nanjing Medical University, Jiangsu Province Hospital, Nanjing 210029, China; ^4^ Department of Oncology, People's Hospital of Jiangdu, Yangzhou 225200, Jiangsu, China

**Keywords:** large granular lymphocyte, lymphocytosis, transplantation

## Abstract

Post-transplant lymphoproliferative disorders (PTLD) represent a heterogeneous group of diseases that occur following transplantation. Large granular lymphocytic (LGL) lymphocytosis is one type of PTLD, ranging from reactive polyclonal self-limited expansion to oligo/monoclonal lymphocytosis or even to overt leukaemia. LGL lymphocytosis in transplant recipients may present as a relatively indolent version of the condition and may be more common than reported, but its natural history and clinical course have not been well described, and the lack of a reliable classification system has limited studies on this disease. Patients with unexplained cytopenias, autoimmune manifestations, or unexpected remissions may be mislabelled. The purpose of this review was to evaluate the clinical features, immunophenotypes, etiopathogenesis, diagnosis, outcomes and treatment of post-transplantation LGL lymphocytosis. In conclusion, LGL lymphocytosis is a frequent occurrence after transplantation that correlates with certain procedural variables and post-transplant events. LGL lymphocytosis should be considered in patients with unexplained lymphocytosis or when pancytopenia develops after transplantation. The diagnosis of LGL lymphocytosis requires a demonstration of monoclonality, but clonality does not indicate malignancy. Additional studies are necessary to further delineate the potential effects of large granular lymphocytes in the long-term prognosis of post-transplant patients.

## INTRODUCTION

Post-transplant lymphoproliferative disorders (PTLD) represent a heterogeneous group of diseases that occur following transplantation. Most of the diseases are of the B-cell origin associated with the Epstein-Barr virus (EBV) [1, 2]. The overall incidence of PTLD is relatively low (1–20%) [1, 3], but the incidence has risen over the past several years, and now PTLD is the second most common post-transplant malignancy after skin cancers in adults and the most common one in children [4]. Large granular lymphocytic (LGL) lymphocytosis is one type of PTLD and has been reported following autologous and allogeneic haematopoietic stem cell transplantation (SCT), bone marrow transplantation (BMT) and solid organ transplantation (SOT), ranging from reactive polyclonal self-limited expansion to oligo/monoclonal lymphocytosis or even to overt leukaemia [5–8]. LGL lymphocytosis in transplant recipients may present as a relatively indolent version of the disease and may be more common than reported, but its natural history and clinical course have not been well described. Physicians and haematopathologists should be aware of this disease because patients with unexplained cytopenias, autoimmune manifestations, or unexpected remissions may be mislabelled. The small number of patients with LGL lymphocytosis in each transplant centre and the lack of a reliable classification system have limited studies on this disease. Therefore, we aimed to evaluate the clinical features, immunophenotypes, etiopathogenesis, diagnosis, outcomes and treatment of LGL lymphocytosis after transplantation.

### Occurrence

Most studies report that LGL lymphocytosis develops in approximately 0.5% to 18.4% of patients after transplantation, as shown in Table 1. LGL lymphocytosis after SCT is not a rare finding, and the incidence was 18.4% (77/418) in one report [9]. In another report, LGL lymphocytosis appeared in 14 out of 215 patients (7%) after SCT [10]. Mohty M et al. observed LGL lymphocytosis in 6 patients (3%) out of 201 consecutive patients after SCT [5]. In the latest study, Muñoz-Ballester J et al. reported 14 patients (9%) with LGL expansion after SCT in a cohort of 154 patients [12]. In the SOT setting, T-LGL expansion is more frequent and was reported in 18 of 23 SOT recipients [6]. In contrast, Gill H reported that the frequency of T-LGLL was low in post-SCT patients (0.5%, 8/1675) [11].

**Table 1:** Summary of articles about LGL lymphocytosis after transplantation

No. of patients	Gender(M:F)	Median age (years)	Transplantation	Rate of LGL lymphocytosis (patients)	Type of LGL lymphocytosis	Median interval from transplantation to LGL lymphocytosis	Median number of LGL (10^9^/L)	Reference
201	3:3	54 (28–60)	SCT	3% (6)	T-LGL lymphocytosis	295 days (range, 75–450 days)	2.3 (range 2.0–4.1)	[5]
23	NR	NR	SOT	61% (14)	T-LGLL	4 months (range 1.5–15 months)	NR (range 2.2–5.6)	[6]
418	39:38	47 (19–68)	SCT	18.4% (77)	LGL lymphocytosis	312 days (range, 26–1840)	1.6 (range 0.6–2.7)	[9]
215	7:7	38.5 (25–64)	SCT	7% (14)	T-LGL lymphocytosis	16 months (range 3–58 months)	2.1 (range 1.3–11.5)	[10]
1675	4:3	50 (29–81)	SCT	0.5% (8)	T-LGLL	1 month (range 1–8 months)	2.7 (range1.6–4.7)	[11]
154	11:3	48.5 (35–66)	SCT	9.1% (14)	T-LGLL	NR	3.0(range 1.6–6.0)	[12]

NR, not reported.

The interval between transplantation to LGL lymphocytosis ranges from 1 to 61 months, and the LGL count ranges from 0.6 to 11.5 × 10^9^/L (Table 1). We defined patients with or without LGL lymphocytosis as LGL^+^ patients or LGL^−^ patients, respectively. Wolniak KL et al. evaluated LGL lymphocytosis in myeloma patients after SCT compared with patients without SCT [13]. The incidence of LGL lymphocytosis increased in patients following SCT compared with patients without SCT. The patients had an average LGL count of 0.59 × 10^9^/L (range, 0.16–3.23 × 10^9^/L) compared with 0.20 × 10^9^/L (range, 0.05–0.34×10^9^/L) in patients without SCT. LGLs constituted an average of 38% (range, 19%–73%) of the lymphocytes in the peripheral blood (PB) of the transplant patients and 12% (range, 3–15%) of the lymphocytes in patients without transplantation.

### Possible mechanisms

Does LGL lymphocytosis represent either a secondary cancer or a clonal LGL aberration or does it derive from the expansion of coexisting, undetected small-sized LGL clones? What triggers the expansion of LGLs after transplantation? The pathogenesis of LGL lymphocytosis, although not properly elucidated thus far, seems to be multifactorial. Most studies have suggested a causal relationship with chronic antigenic stimulation due to viral infection, autoimmune diseases, graft-versus-host disease (GVHD), or immunosuppression with decreased immunosurveillance, leading to LGL lymphocytosis or neoplastic transformation [14, 15]. The proliferation is initially polyclonal, but it often becomes unrestricted, perhaps due to “second hits” such as cytomegalovirus (CMV) infection [10], and then becomes monoclonal or malignant.

### Chronic antigenic stimulation

LGL lymphocytosis after transplantation may be associated with long-term antigenic stimulation due to GVHD or viral infections, especially CMV. A higher number of serious CMV reactivation events occur in the early post-transplant period in patients with LGL lymphocytosis. After transplantation, EBV serologic status, CMV reactivations, and viral infections may represent a pathologic state in which chronic antigenic stimulation may result in LGL lymphocytosis.

### Viral infection

LGL lymphocytosis is more frequent following viral infection and is likely to be the result of long-term stimulation by viral antigens. Some viruses, such as CMV, are associated with the expansion of LGLs. Chronic CMV antigen stimulation has been postulated as a potential driver for LGL lymphocytosis as its reactivation has been consistently associated with LGL lymphocytosis [5, 10]. There are several reports of nonclonal T-LGL lymphocytosis after transplantation, usually in response to CMV infections [5, 14–18].

CMV infection showed a significant association with T-LGL expansion. At the time of CMV infection, no abnormal lymphoid cells were seen on PB smears. The median time between CMV infection and LGL expansion was 336 days (range, 66–339), but at the time of LGL lymphocytosis diagnosis, none of the patients had concurrent CMV infection [5]. Although the median time between CMV infection and LGL expansion diagnosis was long, it is possible that latent LGL expansion could have initiated after CMV infection but before diagnosis. CMV-IgG seropositivity was greater in LGL^+^ patients (60/76, 79%) than in LGL^−^ patients (173/339, 51%) [9]. CMV-IgG seropositivity indicates that a person was infected with CMV at some time, but does not necessarily represent recent CMV reactivation.

LGL^+^ patients presented with a history of CMV reactivation more often than LGL^−^ patients [19]. A report demonstrated that 9 out of 14 LGL^+^ patients (64%) showed CMV reactivation, but only 43 out of 201 LGL^−^ patients (24%) exhibited CMV reactivation [10]. CMV reactivation may be triggered by immunosuppressive therapy, subsequently leading to monoclonal or oligoclonal LGL expansions. CMV reactivation was a common event after SCT and LGL lymphocytosis implicated CMV reactivation as a related factor. This finding is consistent with the hypothesis that LGL lymphocytosis could be related to CMV.

These results indicate that most patients have clinical histories of CMV infection throughout their lives, but it is unlikely that they acquired a recent infection or experienced recent CMV reactivation. Additional studies are necessary to address the potential role of CMV in the pathogenesis of LGL lymphocytosis.

EBV-DNA was not detected in the recipients with T-LGLL, and T cells do not usually express EBV receptor CD21; therefore, EBV infection may not have a role in T-LGLL after transplantation [6, 20–22]. Sabnani I et al. reported that the PCR results for HTLV1 were negative in SOT recipients with T-LGLL [6].

Viral stimulation can trigger the expansion of LGLs, and such viral infections can stimulate the generation of antigen-specific LGLs with the coincidental generation of virus-specific LGLs; these LGLs were shown to be cross-reactive for allogeneic and virus-infected syngeneic target cells [23].

### Alloantigens from the grafts

In transplant recipients, viruses are not the only source of constant antigenic stimulation; the allograft may represent an additional stimulus. The foreign antigens from grafts are a source of constant antigenic stimulation and serve as a driving force of LGL expansion. Recipient-derived LGLs may expand in response to the mismatched alloantigens expressed by the haematopoietic cells of the donors. In the setting of SOT, as with allogeneic SCT, constant alloantigenic stimuli can elicit LGL expansion. Long-term stimulation by alloantigens may be the underlying aetiology of LGL lymphocytosis. In the allogeneic transplant series, LGLs expanded in response to foreign antigens. Interestingly, LGL lymphocytosis has also been reported in autologous SCT [24–27], in which allogeneic foreign antigens are not present, suggesting another mechanism as the driving force of LGL lymphocytosis.

### GVHD

LGL lymphocytosis following transplantation may be related to chronic antigenic stimulation due to GVHD and is significantly more frequent in patients who developed GVHD. Kim D et al. reported a strong association of LGL lymphocytosis with chronic GVHD [9]. In another study, LGL^+^ patients presented more often with a history of acute GVHD compared with LGL^−^ patients [10]. In a recent report, LGL lymphocytosis was significantly associated with the development of chronic GVHD (13/14 patients) [12], but some studies have shown no association between GVHD and LGL expansion [25]; therefore, the observed data in the literature are inconsistent.

### Immunosuppressive therapy

Immunosuppressive therapy after transplantation is one cause of LGL lymphocytosis. An undesirable effect of immunosuppression is the loss of innate defence mechanisms that prevent the development of lymphoproliferative disease [1, 7, 8]. Immunosuppressive therapy can also result in CMV reactivation, subsequently leading to LGL lymphocytosis. The intensity of immunosuppression and the additional genotoxic stress from therapeutic regimens may represent further risk factors.

LGL lymphocytosis after transplantation was significantly more frequent in patients who received a reduced preparative regimen compared to the conventional myeloablative regimen. Mohty M et al. reported that T-LGL lymphocytosis occurred more frequently after a reduced preparative regimen [5]. LGL lymphocytosis occurred more frequently following a reduced preparative regimen (4/49 patients) than after a myeloablative regimen (2/152 patients) [5]. There are two explanations for this phenomenon: a reduced preparative regimen may lead to a state of immune balance that favours the emergence of LGLs. Alternatively, a reduced preparative regimen may allow engraftment with minimal procedure-related toxicity, but this setting is associated with an increased rate of viral infections [28–32]. Currently, the use of reduced preparative regimens is rapidly increasing, and awareness is important because patients with unexplained cytopenias, autoimmune manifestations, or unexpected remissions may be mislabelled.

### Donor-derived T-LGL clones

The rare occurrence of the passage of tumour cells from donors to recipients has been described after SOT [33], and even healthy donors with normal-appearing cells and differential counts can harbour malignant neoplasms. Donor-derived T-LGLL was first reported after allogeneic BMT for chronic myeloid leukaemia, and an analysis of donor-recipient DNA chimerism showed that the marrow (containing 50% LGLs) and PB (containing 80% LGLs) were of donor origin [34]. A case of donor-derived T-LGLL was reported in a 16-year-old boy who received allogeneic SCT for peripheral T-cell lymphoma that was not otherwise specified [35]. The patient presented with persistent neutropenia and splenomegaly 9 months after SCT when the chimerism study showed a 100% donor pattern. Post–allogeneic BMT T-LGLs with B cell lymphoproliferative disorders were reported in 2 patients and were of donor origin as determined by chimerism studies. It is likely that a combination of chronic antigenic stimulation and immunosuppression led to the neoplastic transformation of a donor-derived T-cell population. Another possibility is the transfer of a neoplastic T-cell clone from the donor to the recipient. It would be useful to examine the donor to exclude the occult presence of clonal LGL proliferation.

### Molecular mechanism

Genetic changes may lead to the formation of LGL lymphocytosis. LGLL has been shown to have increased signal transducers and activators of transcription 3 (STAT3) activity, and aberrant STAT3 signalling underlies the pathogenesis. Because of pathogenetic and clinical similarities, LGL lymphocytosis after transplantation may also involve activation of the STAT3 pathway. However, studies evaluating this pathway in SCT-associated cases are lacking. STAT3 mutations have been demonstrated in both T-cell- and NK cell-derived cases and are associated with increased phosphorylated STAT3 protein (pSTAT3), which can be visualized by immunohistochemistry (IHC) [36, 37]. In a report, IHC was used to assess the nuclear localization of pSTAT3 in seven patients who developed LGLL following SCT [37]. More recently, Liang CS et al. showed that pSTAT3 was expressed in SCT-associated T-LGLL by IHC [38]. As a result, specific STAT3 inhibitors have been evaluated as novel therapeutic agents for the treatment of T-LGLL [39]. An IHC stain for pSTAT3 showed positive nuclear staining in a subset of LGLs ranging from 10 to 20% of the infiltrate in 5 of 6 patients (83%). Two patients had a second sample available for testing and both samples were positive for pSTAT3, which has been associated with activation of the STAT3 pathway and STAT3 mutations [35, 36].

STAT3 mutations resulting in the persistent proliferation of LGLs are frequent in LGLL [36, 37, 40]. No STAT3 mutation could be found in the CD3^+^ sorted fraction of LGL^+^ patients with long-term post-allo SCT surveillance. Hidalgo Lopezet JE et al. performed a mutation analysis for STAT3, but no mutation was detected in either pre-SCT or post-SCT specimens [35]. Such cases share a similar pathogenesis to non-transplant-associated cases and should be viewed as true neoplastic disease. Finally, previous reports have shown increased apoptosis in leukaemic LGLs treated with agents targeting the STAT3 pathway, and we recommend similar studies in SCT-associated cases.

### Diagnosis

The diagnosis of LGL lymphocytosis is still challenging due to several features of the condition: (1) a relatively low frequency; (2) only a slight increase in WBC count; (3) the morphological resemblance among normal LGLs, reactive LGLs and leukaemic cells; and (4) the cytopenia associated with LGL lymphocytosis can be attributable to other complications of transplantation. Therefore, this disease may be missed or interpreted as recurrence of the initial disease. Based on the published literature, there are several important considerations for a diagnosis of LGL lymphocytosis after transplantation: (1) a PB lymphocyte count equal to or greater than 3.0 × 10^9^/L and an absolute LGL count equal to or greater than 1.5 × 10^9^/L for at least 6 months; (2) the predominance of LGLs in PB smear samples; (3) emergence after transplantation; (4) the phenotypes of the LGLs, which are either CD3^−^CD56^+^ NK-cells or CD3^+^CD8^+^ T-cells; and (5) the clonal nature of LGL lymphocytosis. Recently, STAT3 mutations have been shown to have diagnosticvalue in distinguishing T-LGLL from other mature T-cell neoplasms and reactive conditions [36, 37]. Because of the pathogenetic and clinical similarities, LGL lymphocytosis warrants screening for STAT3 mutations in LGL^+^ patients after transplantation. In SCT-associated LGLL, STAT3 activation can help identify patients with LGLL versus reactive LGL expansion and is useful for both diagnostic and therapeutic purposes.

**Figure 1:**
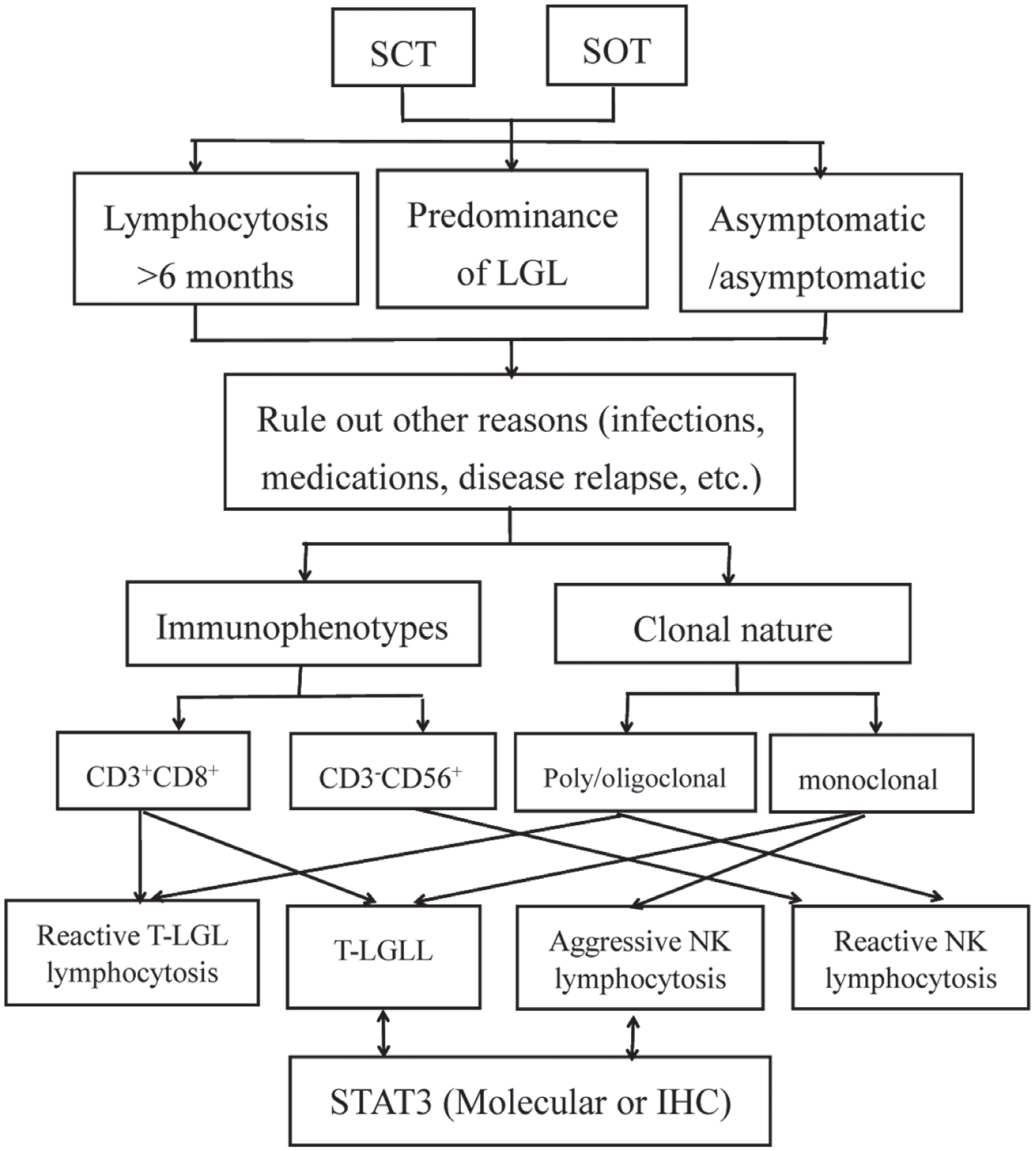
Diagnostic algorithm for the differential diagnosis of LGL lymphocytosis after transplantation Abbreviations: IHC, immunohistochemistry; SCT, stem cell transplantation; SOT, solid organ transplantation; T-LGL, T-cell large granular lymphocytic; TCR, T-cell receptor.

It is important that haematopathologists and haematologists are aware of this disease so they can treat it. We propose a diagnostic algorithm for the diagnoses of LGL lymphocytosis after transplantation (Figure 1).

### Clinical features

The patients with LGL lymphocytosis were all adults between the age of 19 to 81 years, and all viral serological tests were negative (Table 1). LGL lymphocytosis after transplantation has a variable clinical course, ranging from indolent behaviour to refractory cytopenias. Some patients with T-LGLL after transplantation showed a chronic, indolent course that was not related to the primary disease. LGL counts remained stable for long periods without evidence of disease progression or organ infiltration. Therefore, the clinical presentation was similar to de novo T-LGLL, suggesting that a similar pathological process may be involved. We compared the clinicopathological characteristics between T-LGLL after transplantation and de novo T-LGLL (Table 2).

**Table 2:** The contrast of the clinicopathological characteristics between T-LGLL after transplantation and de novo T-LGLL

	Median age (years)	Male:Female	Clinical features	Phenotype	Treatment	STAT3 mutations	Associated virus
de novo T-LGLL	60	1:1	Asymptomatic;symptomatic with cytopenia or autoimmune conditions(RA, PRCA, ITP, etc.)	CD3^+^CD4^+/−^ CD8^+^CD16^+^CD56^−^CD57^+^TCRαβ^+^ (10% are TCRγδ^+^)	Observation or immunosuppressive regimen	Yes	HTLV
T-LGLL after transplantation	50 [11] 48.5 [12]	4:3 [11] 11:3 [12]	Similar to de novo T-LGLL, less prevalent autoimmune disease, and emergence after transplantation	CD3^+^CD4^−^CD8^+^CD16^+^CD26^−^CD28^−^CD56^−^CD57^+^TCRαβ^+^TCRγδ^−^	Observation or reduced immunosuppression	No	CMV

Abbreviations: RA, rheumatoid arthritis; PRCA, pure red cell aplasia; ITP, immune thrombocytopenia; HTLV, human T-cell leukemia/lymphoma virus; CMV, cytomegalovirus.

Some patients with LGL lymphocytosis had relevant clinical signs. Sabnani I et al. reported that fatigue was the most common manifestation, presenting in 30% of patients [6]. Another common symptom was mild to severe cytopenia. Anaemia and thrombocytopenia were also frequent findings. Anaemia was seen in 3 out of 4 renal transplant patients and in 1 out of 10 cardiac transplant patients [6]. Thrombopenia was observed in 3 out of 6 patients (50%), but no severe haemorrhagic events were diagnosed [5]. Nann-Rütti S et al. reported that 8 out of 14 patients (57%) showed mild hyporegenerative anaemia, and thrombocytopenia was present in 2 of the 14 patients (14%) [10]. Neutropenia was present in 4 out of 7 patients, and 2 patients experienced severe neutropenia associated with septicaemia [5].

Cytopenias were considered to be linked to LGL lymphocytosis for 2 reasons: (1) LGLs can target haematopoiesis, inducing mild to severe cytopenia, and (2) cytopenias are considered to be linked to transplant-related problems rather than LGL expansion. Cytopenias were detected in some patients, all in the early stages after haematopoietic engraftment. During the follow-up period, the cytopenias gradually resolved despite the persistence of LGL lymphocytosis, suggesting that the initial cytopenias were related to instability of the haematopoietic engraftment during the early stages of SCT rather than haematopoietic suppression mediated by the LGLs.

Additional clinical symptoms include several autoimmune phenomena, particularly rheumatoid arthritis, pure red cell aplasia, autoimmune thrombocytopenia, autoimmune haemolytic anaemia, or polymyositis [5]. A subset of patients may develop LGLL with potentially significant symptomatology and mortality after SCT. We summarised the clinical features of the patients with T-LGLL after SOT in Table 3.

**Table 3:** Clinical features of the patients with T-LGLL after SOT

Initial diagnosis	Age (y)	Gender	Transplant Organ	Virus	After transplantation (mo)	Hemoglobin, (g/L)	WBC count, (× 109/L)	Platelet count, (× 109/L)	LGLs, (× 109/L)	Treatment	Outcome	References
Cirrhosis	40	M	Liver	EBV-	3–4	-	-	-	1.4	-	-	[46]
ESRD	44	M	Kidney	CMV, EBV, HTLV-	5	11.9	6.3	95	1.1	NO	-	[47]
ESRD	37	M	Kidney	CMV, EBV, HTLV-	133	6.3	12.4	376	5.2	CCP	PR	
ESRD	69	F	Kidney	CMV, EBV, HTLV-	15	10.2	11	210	3.6	-	-	
-	36	F	Kidney	EBV, Parvovirus -.	118	5.8	5.2	50.8	1.6	CCP	CR	[55]
Cirrhosis, nephropathy	69	F	Liver, kidney	EBV, parvovirus-	6	7	7.8	273	5.5	CCP	PR	[56]
FSGS	48	M	Kidney	parvovirus -	14	7	6.8	424	5.1	CCP	PR	
glomerulonephritis	55	M	Kidney	Parvovirus-	10	8	4.8	148	2.7	CCP, RTX, ATG	NR	
FSGS	46	M	Kidney	EBV -	12	7.3	6.4	302	-	CCP, MTX, ATG	NR	
ESRD	47	M	Kidney	CMV,EBV-	144	10.5	16.7	248	-	-	-	[57]

Abbreviations: ESRD, End-stage renal disease; FSGS, focal segmental glomerulosclerosis;

CCP, oral cyclophosphamide; RTX, rituximab; ATG, antithymocyte globulin; MTX, oral methotrexate.

### Immunophenotypes

The immunophenotypes of LGL lymphocytosis after transplantation are CD3^+^CD8^+^ cytotoxic T-cells and CD3^−^CD16^+^/56^+^ NK-cells, and the CD3^+^CD8^+^ phenotype is present in most cases. LGLs are commonly positive for CD2, CD3 CD5, CD7 and CD8, CD16, CD57 and TCRαβ and negative for CD4, CD16, CD26, CD28, CD56, and TCRγδ [5, 10–12]. LGL lymphocytosis can display a heterogeneous phenotype. In some patients, CD3^+^CD8^+^ LGLs were negative to slightly positive for CD5, CD56, CD62L and CD94, but TIA-1 are expressed [10–13, 41]. CD3^+^CD8^+^ LGLs displayed late activation markers, including HLA-DR and CD57, but are negative for early activation markers, including IL-2 receptor CD25 and transferrin receptor CD71 [41]. CD3^+^CD8^+^ LGLs displayed CD11c, CD38, CD45RA, CD69 and adhesion molecules CD54 and CD58 but were negative for CD45RO. Strong heterogeneity was observed for CD28 expression, with ranges from 0 to 41% [41].

### Clonality

The critical diagnostic decision in the evaluation of LGL lymphocytosis is whether the expansion is neoplastic or reactive. Since the LGLL immunophenotype overlaps somewhat with that of nonclonal LGLs, establishing a diagnosis of LGLL may rely on clonal TCR gene rearrangement or CD158/killer cell immunoglobulin-like receptor (KIR) expression. TCR gene rearrangements are present in myeloma patients regardless of therapy and increase after SCT [13]. TCR gene rearrangements were observed in non-transplant patients, but transplant patients showed clonal TCR gene rearrangements more often than the non-transplant patients (65% vs. 33%, respectively) [13]. The T-LGLL patients had clonal TCR gene rearrangement more often than the patients with reactive LGL lymphocytosis. In patients with reactive LGL lymphocytosis, TCR gene rearrangement was described as clonal, oligoclonal or polyclonal in 77%, 16% and 7% of cases, respectively, but TCR gene rearrangement was clonal in all T-LGLL patients [12]. High frequencies of clonal TCR gene rearrangements were also observed in SOT recipients. Sabnani I et al. reported that 14 of 23 (61%) patients were positive for clonal TCR gene rearrangement, and the incidence was higher in cardiac transplant patients than in renal transplant patients (71% vs. 44%) [6].

In another study, one patient demonstrated KIR restriction, but no expression of KIRs was identified in most other patients. Therefore, an assessment for clonality based on KIR expression remained undefined [13]. However, the KIR panel used for the standard analysis of LGLs examined only 4 members of that family.

STAT3 mutations may serve as useful tools to discriminate malignant NK/T lymphocytosis from reactive expansions, especially in the establishment of clonality using STAT3 mutations as clonal markers. LGLs from 82 patients with LGL lymphocytosis were assessed for STAT3 mutations, but none were found [12]. Whether STAT3 mutations can be acquired requires further investigation. Even if LGL lymphocytosis is proven to be clonal, we must remember that clonality does not indicate malignancy.

### Types of LGL lymphocytosis

Transplant-associated LGL lymphocytosis typically involves the monoclonal or oligoclonal expansion of cytotoxic T- or NK-cells. Reactive LGL lymphocytosis is usually polyclonal, transient, and asymptomatic. Neoplastic clonal LGL expansions are classified as T-LGLL and NK-cell lymphocytosis [42, 43].

### Reactive LGL lymphocytosis

Reactive LGL lymphocytosis has been observed to occur after SCT and is significantly associated with CMV infection [5, 9, 10, 24]. The lymphocytes appeared at a median of 16 months (range, 3–58 months) after SCT and lasted for a median duration of 31 months (range, 2–179 months) [10]. The most important feature of reactive LGL lymphocytosis that it is polyclonal and may rarely evolve into LGLL. There were no related haematopoietic disturbances, and the patients were asymptomatic, but a syndrome similar to de novo T-LGLL may develop in some patients, with clinical manifestations including haematopoietic suppression and autoimmune phenomena such as polyarthritis and polyclonal hypergammaglobulinemia [14].

### T-LGLL

T-LGLL is the most frequent form of LGL lymphocytosis after transplantation and has been reported in single case reports or in small case series [25, 34, 43, 44, 45]. T-LGLL in post-transplant patients may have a different clinical course and pathogenesis compared with de novo T-LGLL. Gill H et al. reported 7 cases of T-LGLL after BMT [11], and some reports have described T-LGLL following SCT [5, 7, 8]. Similar to T-LGLL after SCT, T-LGLL has been reported in heart, liver and renal transplant patients (Table 3) [46, 47]. None of the patients developed neutropenia or had active donor organ rejection, and most patients did not receive therapy. These LGL expansions have been defined as neoplastic processes because of their clonal nature. Aggressive T-LGLL is an infrequent complication after transplantation. Feher O et al. reported aggressive T-LGLL in a patient following orthotopic liver transplantation [46]. A unique feature of this case was the detection of a chromosomal deletion at 1p32 involving the tal-1 gene, an abnormality previously described only in aggressive T-cell neoplasms. Au WY et al. also reported a case of donor-derived T-LGLL that occurred after SCT and progressed along a fatal course [34]. The patient had concomitant GVHD complicating the clinical picture, but the progressive course suggests that it may have been a neoplastic lymphoproliferative process rather than LGL expansion as seen in the majority of post-transplant patients.

### NK-cell lymphocytosis

NK-cell lymphocytosis is a rare type of LGL lymphocytosis after transplantation and was first reported by Hsi ED et al. [48]. After renal transplantation, a patient developed NK-cell lymphocytosis and the tumour cells expressed features consistent with NK cells, but EBV was absent from the tumour cells. Kwong YL et al. also reported a case of NK-cell lymphocytosis after transplantation [49]. The tumour cells showed EBV infection and were characterized cytologically by LGL morphology, immunophenotypically by the expression of CD2, cytoplasmic CD3 and CD56, genotypically by the T-cell receptor (TCR) gene in the germline configuration and clinically by an aggressive course with a poor prognosis. Sabnani et al. reported 10 cases of NK-cell lymphocytosis after SOT[6]. The significance of NK cell lymphocytosis is that this disorder seems to be highly malignant, and aggressive treatment may be required.

### Treatments

In the non-transplant setting, the indications for T-LGLL treatment are severe neutropenia with recurrent infections, severe refractory anaemia, and severe thrombocytopenia [50]; cyclophosphamide, cyclosporine, and methotrexate have been used as first-line treatment agents [51, 52], and alemtuzumab and antithymocyte globulin have been proposed for refractory disease [53, 54]. There are no formal trials guiding the specific management options for LGL lymphocytosis after transplantation. We should determine whether lesion responds to a decrease in immunosuppression or if it requires lymphoma treatment or immunological therapy.

Reactive LGL lymphocytosis follows an indolent course and does not require therapy. Almost all patients with T-LGLL after transplantation remained stable for years and did not require specific treatment; few patients have shown an aggressive clinical course with progressive pancytopenia and death in a short amount of time. In 6 LGL^+^ patients, 3 patients did not require specific therapy, and 3 patients required therapy. All patients remain alive, with persistent circulating LGLs in 4 cases, and were disease-free with good quality of life by the end of the follow-up [5]. In T-LGLL patients after SOT, oral cyclophosphamide has been used with good results [47, 55, 56]. Two of 4 patients had substantial haematologic responses with oral cyclophosphamide, 3 patients had stable allograft function, and 1 patient lost the allograft due to delayed cellular rejection [56].

Some patients showed minimal responses to several lines of therapy. At the end of the follow-up, 4 patients were alive, including one with persistent disease and three with progressive disease. Three patients died due to complications of disease, including one with persistent disease and two with progressive disease [38].

Chimerism studies to determine the origin of neoplasia that develops after SCT are important for the management of these patients. Malignant neoplasms of recipient but not donor origin may be controllable by enhancing the putative graft-vs-malignancy effect through reduced immunosuppression and the use of cytokines such as IL-2 [34]. LGLL in SOT recipients has a variable response to chemotherapeutic agents. Treatment agents to eliminate these clones were associated with treatment failure. Such findings may help explain why some cases of SCT-associated LGLL show higher rates of symptomatology and treatment resistance.

A reduction in immunosuppression is advisable in recipients with T-cell PTLD [57, 58], but there is no evidence to support this strategy in LGLL. Most of these patients are usually on post-transplant immunosuppressive therapy, such as cyclosporine and prednisone, which is considered effective therapy for LGLL in the non-transplant setting. It is interesting that LGL expansion occurs despite ongoing treatment with immunosuppressive medications that are normally used to treat LGL disorders.

Constitutive STAT3 activation has been demonstrated in LGL lymphocytosis. Higher levels of STAT3 are linked to tumourigenesis and chemoresistance. Therefore, the expression levels of STAT3 can be used as predictive markers for therapeutic responses, and down-regulation of STAT3 is a very attractive treatment strategy.

### Favourable response

There is no clear evaluation for the potential positive or negative effects of LGL lymphocytosis, and whether these expansions represent true post-transplant neoplastic processes, immune responses, or a combination of both is not clear. A T/NK-cell origin in a PTLD is considered an adverse prognostic indicator, but LGL lymphocytosis exhibits a benign chronic course and patients with LGL lymphocytosis showed significantly better survival than patients with other PTLDs, such as peripheral T-cell lymphoma, unspecified or hepatosplenic T-cell lymphoma [59]. Whether LGL lymphocytosis was clonal or nonclonal, transplant-associated LGL lymphocytosis should be viewed as a potentially beneficial immune response rather than a true neoplastic process. T-LGL lymphocytosis is reported to expand in patients after SCT and is thought to be a good prognostic sign, indicating a lower relapse rate compared with LGL^−^ patients [9]. Haji S et al. reported that LGLs may contribute to disease control in the first report demonstrating that EBV-specific LGLs were spontaneously induced after SCT [60]. After SCT, donor-derived EBV-specific LGLs increased and tumours regressed soon after LGL expansion and then worsened again when the LGLs disappeared. When patient LGLs were incubated with EBV-transformed lymphoblastoid cell lines, the EBV-specific LGLs had a tumour-killing effect *in vitro*. The OS of LGL^+^ patients was longer than LGL^−^ patients (no median survival time versus 132 months), and none of the 14 LGL^+^ patients relapsed, but 31/140 LGL^−^ patients (22%) did [12]. In patients with chronic lymphocytic leukaemia after SCT, the presence of LGLs was related to event-free survival or OS, and none of the LGL^+^ patients relapsed [61]. LGL lymphocytosis was confirmed as an independent favourable prognostic factor for OS and non-relapse mortality (NRM). The presence of increased LGLs was associated with improved OS (86.2 months vs 53.8 months, *P* < 0.001), lower NRM (3.2% vs 27.3%, *P* < 0.001) and a lower relapse rate (9.6% vs 29.4%, *P* < 0.001) [9].

LGL lymphocytosis has been related to an anticancer effect. LGLs may have similar properties to effector lymphocytes and may mediate the graft-versus-malignancy effect. *In vitro*, LGLs can mediate cytolytic activity on tumour cells. In *vivo*, the timing of LGL lymphocytosis following transplantation was associated with sustained complete molecular remission [15]. These observations are consistent with earlier studies on polyclonal T-LGL proliferation that reported favourable outcomes for the original malignancies.

The immune reactions between donor-derived immunocompetent T lymphocytes and host-type tumour cells are considered the major source of anti-tumour effects. First, donor-derived LGLs show cross-reactivity against recipient tumour cells. Donor-derived LGLs constitute a crucial component of the successful treatment of relapses following SCT [62]. Second, LGLs are equipped with different costimulatory molecules that may play a role in the cytotoxic mechanisms of LGLs [63]. Third, LGLs can be involved in graft-versus-host reactions and mediate cytotoxic activity against neoplastic cells, and the long-term complete remission achieved by some high-risk patients occurred with or following LGL expansion [39, 64]. Fourth, viral infections can elicit LGL expansion against tumour cells both *in vitro* and *in vivo*, and LGLs can mediate killer cell activity or develop peptide-specific cytotoxicity [65].

LGL lymphocytosis after SCT does not always represent a good prognosis. In some cases, the clinical courses were indolent, and LGL lymphocytosis did not affect prognosis [22, 23]. In one report, LGL lymphocytosis of recipient origin may have contributed to late graft rejection by attacking donor stem cells [66]. When LGL expansion occurs, the origin of the LGLs should be determined as soon as possible, and an appropriate intervention, such as the discontinuation of immunosuppressants and donor lymphocyte infusions, may be considered.

## CONCLUSIONS

LGL lymphocytosis is a frequent occurrence after transplantation that correlates with certain procedural variables and post-transplantation events and should be considered in patients with unexplained lymphocytosis or when pancytopenia develops after transplantation. Not all LGL lymphocytosis is clonal, so determining a diagnosis of LGL lymphocytosis requires a demonstration of clonality; however, clonality does not indicate malignancy. Finally, additional studies are necessary to further delineate the potential effects of LGLs in the long-term prognosis of post-transplant patients. However, comparisons of these studies are limited by unavoidable heterogeneity in terminology, diagnostic criteria and patient populations.

## References

[1] Taylor AL, Marcus R, Bradley JA (2005). Post-transplant lymphoproliferative disorders (PTLD) after solid organ transplantation. Crit Rev Oncol Hematol.

[2] Allen UD, Preiksaitis JK (2013). Epstein-Barr virus and posttransplant lymphoproliferativedisorder in solid organ transplantation. Am J Transplant.

[3] Dierickx D, Tousseyn T, Sagaert X, Fieuws S, Wlodarska I, Morscio J, Brepoels L, Kuypers D, Vanhaecke J, Nevens F, Verleden G, Van Damme-Lombaerts R, Renard M (2013). Single-center analysis of biopsy-confirmed posttransplant lymphoproliferative disorder: incidence, clinicopathological characteristics and prognostic factors. Leuk Lymphoma.

[4] Gallego S, Llort A, Gros L, Sanchez de Toledo J, Bueno J, Moreno A, Nieto J, Sanchez de Toledo J (2010). Post-transplant lymphoproliferative disorders in children: the role of chemotherapy in the era of rituximab. Pediatr Transplant.

[5] Mohty M, Faucher C, Vey N, Chabannon C, Sainty D, Arnoulet C, Gaugler B, Gastaut JA, Maraninchi D, Olive D, Blaise D (2002). Features of large granular lymphocytes (LGL) expansion following allogeneic stem cell transplantation: a long-term analysis. Leukemia.

[6] Sabnani I, Zucker MJ, Tsang P, Palekar S (2006). Clonal T-large granular lymphocyte proliferation in solid organ transplant recipients. Transplant Proc.

[7] Sabnani I, Tsang P (2007). Are clonal T-cell large granular lymphocytes to blame for unexplained haematological abnormalities?. Br J Haematol.

[8] Isobe T, Tanimoto TE, Nakaji G, Miyamoto T, Yamasaki S, Takase K, Numata A, Fukuda T, Nagafuji K, Inaba S, Harada M (2005). Autoimmune thrombocytopenia with clonal expansion of CD8-positive T-cells after autologous peripheral blood stem cell transplantation for diffuse large B-cell lymphoma. Bone Marrow Transplant.

[9] Kim D, Al-Dawsari G, Chang H, Panzarella T, Gupta V, Kuruvilla J, Lipton JH, Messner HA (2013). Large granular lymphocytosis and its impact on long-term clinical outcomes following allo-SCT. Bone Marrow Transplant.

[10] Nann-Rütti S, Tzankov A, Cantoni N, Halter J, Heim D, Tsakiris D, Arber C, Buser A, Gratwohl A, Tichelli A, Rovó A (2012). Large granular lymphocyte expansion after allogeneic hematopoietic stem cell transplant is associated with a cytomegalovirus reactivation and shows an indolent outcome. Biol Blood Marrow Transplant.

[11] Gill H, Ip AH, Leung R, So JC, Pang AW, Tse E, Leung AY, Lie AK, Kwong YL (2012). Indolent T-cell large granular lymphocyte leukaemia after haematopoietic SCT: a clinicopathologic and molecular analysis. Bone Marrow Transplant.

[12] Muñoz-Ballester J, Chen-Liang TH, Hurtado AM, Heras I, de Arriba F, García-Malo MD, Iniesta P, Lozano ML, Nieto JB, Ortuño FJ, Osma Mdel M, Padilla J, Teruel-Montoya R (2016). Persistent cytotoxic T lymphocyte expansions after allogeneic haematopoietic stem cell transplantation: kinetics, clinical impact and absence of STAT3 mutations. Br J Haematol.

[13] Wolniak KL, Goolsby CL, Chen YH, Chenn A, Singhal S, Jayesh Mehta, Peterson LA (2013). Expansion of a clonal CD8+CD57+ large granular lymphocyte population after autologous stem cell transplant in multiple myeloma. Am J Clin Pathol.

[14] Dolstra H, Preijers F, Van de Wiel-van Kemenade E, Schattenberg A, Galama J, de Witte T (1995). Expansion of CD8+CD57+ T cells after allogeneic BMT is related with a low incidence of relapse and with cytomegalovirus infection. Br J Haematol.

[15] Mohty M, Faucher C, Gaugler B, Vey N, Sainty D, Arnoulet C, Mozziconacci MJ, Isnardon D, Gastaut JA, Maraninchi D, Olive D, Blaise D (2001). Large granular lymphocytes (LGL) following non-myeloablative allogeneic bone marrow transplantation: a case report. Bone Marrow Transplant.

[16] Wang EC, Moss PA, Frodsham P, Lehner PJ, Bell JI, Borysiewicz LK (1995). CD8highCD57+ T lymphocytes in normal, healthy individuals are oligoclonal and respond to human cytomegalovirus. J Immunol.

[17] Delobel P, Godel A, Thebault S, Alric L, Duffaut M (2006). Transient clonal expansion of T-large granular lymphocytes during primary cytomegalovirus infection. J Infect.

[18] Rossi D, Franceschetti S, Capello D, De Paoli L, Lunghi M, Conconi A, Gaidano G (2007). Transient monoclonal expansion of CD8+/CD57+ T-cell large granular lymphocytes after primary cytomegalovirus infection. Am J Hematol.

[19] Dearden C (2011). Large granular lymphocytic leukaemia pathogenesis and management. Br J Haematol.

[20] Hsieh YC, Chang ST, Huang WT, Kuo SY, Chiang TA, Chuang SSA (2013). Comparative study of flow cytometric T cell receptor Vβ repertoire and T cell receptor gene rearrangement in the diagnosis of large granular lymphocytic lymphoproliferation. Int J Lab Hematol.

[21] Hussein K, Maecker-Kolhoff B, Klein C, Kreipe H (2011). Transplant-associated lymphoproliferation. Pathologe.

[22] Loughran TP, Zambello R, Ashley R, Guderian J, Pellenz M, Semenzato G, Starkebaum G (1993). Failure to detect Epstein-Barr virus DNA in peripheral blood mononuclear cells of most patients with large granular lymphocyte leukemia. Blood.

[23] Nahill SR, Welsh RM (1993). High frequency of cross-reactive cytotoxic T lymphocytes elicited during the virus-induced polyclonal cytotoxic T lymphocyte response. J Exp Med.

[24] Lau LG, Tan LK, Salto-Tellez M, Koay ES, Liu TC (2004). T-cell post-transplant lymphoproliferative disorder after hematopoietic stem cell transplantation: another case and a review of the literature. Bone Marrow Transplant.

[25] Narumi H, Kojima K, Matsuo Y, Shikata H, Sekiya K, Niiya T, Bando S, Niiya H, Azuma T, Yakushijin Y, Sakai I, Yasukawa M, Fujita S (2004). T-cell large granular lymphocytic leukemia occurring after autologous peripheral blood stem cell transplantation. Bone Marrow Transplant.

[26] Sajeva MR, Greco MM, Cascavilla N, D’Arena G, Scalzulli P, Melillo L, Minervini MM, Bonini A, Di Mauro L, Carotenuto M, Musto P (1996). Effective autologous peripheral blood stem cell transplantation in plasma cell leukemia followed by T-large granular lymphocyte expansion: a case report. Bone Marrow Transplant.

[27] Maggi F, Ricci V, Bendinelli M, Nelli LC, Focosi D, Papineschi F, Petrini M, Paumgardhen E, Ghimenti M (2008). Changes in CD8+57+ T lymphocyte expansions after autologous hematopoietic stem cell transplantation correlate with changes in torquetenovirus viremia. Transplantation.

[28] Mohty M, Faucher C, Vey N, Stoppa AM, Viret F, Chabbert I, Chabannon C, Bouabdallah R, Ladaique P, Collet L, Zandotti C, Maraninchi D, Blaise D (2000). High rate of secondary viral and bacterial infections in patients undergoing allogeneic bone marrow minitransplantation. Bone Marrow Transplant.

[29] Slavin S, Nagler A, Naparstek E, Kapelushnik Y, Aker M, Cividalli G, Varadi G, Kirschbaum M, Ackerstein A, Samuel S, Amar A, Brautbar C, Ben-Tal O (1998). Nonmyeloablative stem cell transplantation and cell therapy as an alternative to conventional bone marrow transplantation with lethal cytoreduction for the treatment of malignant and nonmalignant hematologic diseases. Blood.

[30] Mohty M, Fegueux N, Exbrayat C, Lu ZY, Legouffe E, Quittet P, Lopez-Martinez E, Latry P, Avinens O, Hertog C, Klein B, Eliaou JF, Rossi JF (2001). Reduced intensity conditioning: enhanced graft-versus-tumor effect following dose-reduced conditioning and allogeneic transplantation for refractory lymphoid malignancies after high-dose therapy. Bone Marrow Transplant.

[31] Badros A, Barlogie B, Morris C, Desikan R, Martin SR, Munshi N, Zangari M, Mehta J, Toor A, Cottler-Fox M, Fassas A, Anaissie E, Schichman S (2001). High response rate in refractory and poor-risk multiple myeloma after allotransplantation using a nonmyeloablative conditioning regimen and donor lymphocyte infusions. Blood.

[32] Morecki S, Gelfand Y, Nagler A, Or R, Naparstek E, Varadi G, Engelhard D, Akerstein A, Slavin S (2001). Immune reconstitution following allogeneic stem cell transplantation in recipients conditioned by low intensity vs myeloablative regimen. Bone Marrow Transplant.

[33] Loh E, Couch FJ, Hendricksen C, Farid L, Kelly PF, Acker MA, Tomaszewski JE, Malkowicz SB, Weber BL (1997). Development of donor-derived prostate cancer in a recipient following orthotopic heart transplantation. JAMA.

[34] Au WY, Lam CC, Lie AK, Pang A, Kwong YL (2003). T-cell large granular lymphocyte leukemia of donor origin after allogeneic bone marrow transplantation. Am J Clin Pathol.

[35] Hidalgo Lopez JE, Yabe M, Carballo-Zarate AA, Wang SA, Jorgensen JL, Ahmed S, Lee J, Li S, Schlette E, McDonnell T, Miranda RN, Medeiros LJ, Bueso-Ramos CE (2016). Donor-derived T-cell large granular lymphocytic leukemia in a patient with peripheral T-cell lymphoma. J Natl Compr Canc Netw.

[36] Koskela HL, Eldfors S, Ellonen P, van Adrichem AJ, Kuusanmäki H, Andersson EI, Lagström S, Clemente MJ, Olson T, Jalkanen SE, Majumder MM, Almusa H, Edgren H (2012). Somatic STAT3 mutations in large granular lymphocytic leukemia. N Engl J Med.

[37] Jerez A, Clemente MJ, Makishima H, Koskela H, Koskela H, Leblanc F, Peng Ng K, Olson T, Przychodzen B, Afable M, Gomez-Segui I, Guinta K, Durkin L (2012). STAT3 mutations unify the pathogenesis of chronic lymphoproliferative disorders of NK cells and T-cell large granular lymphocyte leukemia. Blood.

[38] Liang CS, Quesada AE, Goswami M, Johnston PK, Brown RE, Jaso JM (2016). Phosphorylated STAT3 expression in hematopoietic stem cell transplant-associated large granular lymphocytic leukemia. Bone Marrow Transplant.

[39] Bilori B, Thota S, Clemente MJ, Patel B, Jerez A, Afable Ii M, Maciejewski JP (2015). Tofacitinib as a novel salvage therapy for refractory T-cell large granular lymphocytic leukemia. Leukemia.

[40] Rajala HL, Olson T, Clemente MJ, Lagström S, Ellonen P, Lundan T, Hamm DE, Zaman SA, Lopez Marti JM, Andersson EI, Jerez A, Porkka K, Maciejewski JP (2015). The analysis of clonal diversity and therapy responses using STAT3 mutations as a molecular marker in large granular lymphocytic leukemia. Haematologica.

[41] Mollet L, Fautrel B, Leblond V, Bergeron F, Merle-Béral H, Baumelou E, Hubert P, Debré P, Autran B (1999). Leukemic CD3+ LGL share functional properties with their CD8+CD57+ cell counterpart expanded after BMT. Leukemia.

[42] Chan WC, Foucar KM, Morice WG, Catovsky D (2008). T-cell large granular lymphocyte leukaemia. WHO Classiication of Tumours of the Hematopoietic and Lymphoid Tissues.

[43] Wong KF, Yip SF, So CC, Lau GT, Yeung YM (2003). Cytomegalovirus infection associated with clonal proliferation of T-cell large granular lymphocytes: causal or casual?. Cancer Genet Cytogenet.

[44] Chang H, Kamel-Reid S, Hussain N, Lipton J, Messner HA (2005). T-cell large granular lymphocytic leukemia of donor origin occurring after allogeneic bone marrow transplantation for B-cell lymphoproliferative disorders. Am J Clin Pathol.

[45] Kusumoto S, Mori S, Nosaka K, Morita-Hoshi Y, Onishi Y, Kim SW, Watanabe T, Heike Y, Tanosaki R, Takaue Y, Tobinai K (2007). T-cell large granular lymphocyte leukemia of donor origin after cord blood transplantation. Clin Lymphoma Myeloma.

[46] Feher O, Barilla D, Locker J, Oliveri D, Melhem M, Winkelstein A (1995). T-cell large granular lymphocytic leukemia following orthotopic liver transplantation. Am J Hematol.

[47] Gentile TC, Hadlock KG, Uner AH, Delal B, Squiers E, Crowley S, Woodman RC, Foung SK, Poiesz BJ, Loughran TP (1998). Large granular lymphocyte leukaemia occurring after renal transplantation. Br J Haematol.

[48] Hsi ED, Picken MM, Alkan S (1998). Post-transplantation lymphoproliferative disorder of the NK-cell type: a case report and review of the literature. Mod Pathol.

[49] Kwong YL, Lam CC, Chan TM (2000). Post-transplantation lymphoproliferative disease of natural killer cell lineage: a clinicopathological and molecular analysis. Br J Haematol.

[50] Dierickx D, Tousseyn T, Gheysens O (2015). How we diagnose and treat posttransplant lymphoproliferative disorders. Blood.

[51] Qiu ZY, Fan L, Wang R, Gale RP, Liang HJ, Wang M, Wang L, Wu YJ, Qiao C, Chen YY, Xu W, Qian J, Li JY (2016). Methotrexate therapy of T-cell large granular lymphocytic leukemia impact of STAT3 mutation. Oncotarget.

[52] Osuji N, Matutes E, Tjonnfjord G, Grech H, Del Giudice I, Wotherspoon A, Swansbury JG, Catovsky D (2006). T-cell large granular lymphocyte leukemia: a report on the treatment of 29 patients and a review of the literature. Cancer.

[53] Osuji N, Del GI, Matutes E, Morilla A, Owusu-Ankomah K, Morilla R, Dunlop A, Catovksy D (2005). CD52 expression in T-cell large granular lymphocyte leukemia—implications for treatment with alemtuzumab. Leuk Lymphoma.

[54] Bargetzi MJ, Wortelboer M, Pabst T, Franscini L, Gudat H, Tichelli A, Tobler A, Speck B, Gratwohl A (1996). Severe neutropenia in T-large granular lymphocyte leukemia corrected by intensive immunosuppression. Ann Hematol.

[55] Masuda M, Arai Y, Nishina H, Fuchinoue S, Mizoguchi H (1998). Large granular lymphocyte leukemia with pure red cell aplasiain a renal transplant recipient. Am J Hematol.

[56] Kataria A, Cohen E, Saad E, Atallah E, Bresnahan B (2014). Large granular lymphocytic leukemia presenting late after solid organ transplantation: a case series of four patients and review of the literature. Transplantation Proceedings.

[57] Stamatopoulos K, Economidou D, Papadaki T, Vadikolia C, Papathanasiou M, Memmos D, Fassas A (2007). Large granular lymphocyte leukemia after renal transplantation: an immunologic, immunohistochemical and genotypic study. Transplantation.

[58] Rajakariar R, Bhattacharyya M, Norton A, Sheaff M, Cavenagh J, Raftery MJ, Yaqoob MM (2004). Posttransplant T-cell lymphoma: a case series of four patients from a single unit and review of the literature. Am J Transplant.

[59] Leblond V, Dhedin N, Mamzer Bruneel MF, Choquet S, Hermine O, Porcher R, Nguyen Quoc S, Davi F, Charlotte F, Dorent R, Barrou B, Vernant JP, Raphael M (2001). Identification of prognostic factors in 61 patients with posttransplantation lymphoproliferative disorders. J Clin Oncol.

[60] Haji S, Shiratsuchi M, Matsushima T, Takamatsu A, Tsuda M, Tsukamoto Y, Tanaka E, Ohno H, Fujioka E, Ishikawa Y, Imadome KI, Ogawa Y (2017). Achievement of disease control with donor-derived EB virus-specific cytotoxic T cells after allogeneic peripheral blood stem cell transplantation for aggressive NK-cell leukemia. Int J Hematol.

[61] Kollgaard T, Petersen SL, Hadrup SR, Masmas TN, Seremet T, Andersen MH, Madsen HO, Vindeløv L, thor Straten P (2005). Evidence for involvement of clonally expanded CD8+ T cells in anticancer immune responses in CLL patients following nonmyeloablative conditioning and hematopoietic cell transplantation. Leukemia.

[62] Schmid C, Labopin M, Nagler A, Bornhäuser M, Finke J, Fassas A, Volin L, Gürman G, Maertens J, Bordigoni P, Holler E, Ehninger G, Polge E, EBMT Acute Leukemia Working Party (2007). Donor lymphocyte infusion in the treatment of first hematological relapse after allogeneic stemcell transplantationin adults with acute myeloid leukemia: a retrospective risk factors analysis and comparison with other strategies by the EBMT Acute Leukemia Working Party. J Clin Oncol.

[63] Zambello R, Trentin L, Facco M, Siviero M, Galvan S, Piazza F, Perin A, Agostini C, Semenzato G (2000). Analysis of TNF-receptor and ligand superfamily molecules in patients with lymphoproliferative disease of granular lymphocytes. Blood.

[64] Ferrara JL, Guillen FJ, van Dijken PJ, Marion A, Murphy GF, Burakoff SJ (1989). Evidence that large granular lymphocytes of donor origin mediate acute graft-versus-host disease. Transplantation.

[65] Mollet L, Sadat-Sowti B, Duntze J, Leblond V, Bergeron F, Calvez V, Katlama C, Debre P, Autran B (1998). T lymphocytes are enriched in antigen-specific T cells capable of down-modulating cytotoxic activity. Int Immunol.

[66] Nakagawa N, Yamazaki H, Aoki G, Kondo Y, Nakao S (2016). Late Graft rejection in association with T-large granular lymphocyte expansion of recipient origin after human leukocyte antigen-haploidentical stem cell transplantation: a case report. Transplant Proc.

